# Dynamic Changes in Numbers and Properties of Circulating Tumor Cells and Their Potential Applications

**DOI:** 10.3390/cancers6042369

**Published:** 2014-12-16

**Authors:** Ju-Yu Tseng, Chih-Yung Yang, Shu-Ching Liang, Ren-Shyan Liu, Jeng-Kai Jiang, Chi-Hung Lin

**Affiliations:** 1 Institute of Microbiology and Immunology, School of Life Science, National Yang-Ming University, Taipei 11221, Taiwan; E-Mails: magic1304@gmail.com (J.-Y.T.); suejing1@yahoo.com.tw (S.-C.L.); 2 Department of Education and Research, Taipei City Hospital, Taipei 10629, Taiwan; E-Mail: yc3636@hotmail.com; 3 Molecular and Genetic Imaging Core/Taiwan Mouse Clinic, Taipei 11529, Taiwan; E-Mail: rsliu@vghtpe.gov.tw; 4 Biomedical Imaging Research Center, Institute of Clinical Medicine, School of Medicine, National Yang-Ming University, Taipei 11221, Taiwan; 5 National PET/Cyclotron Center, Taipei Veterans General Hospital, Taipei, 11217, Taiwan; 6 Division of Colon & Rectal Surgery, Department of Surgery, Taipei Veterans General Hospital, Taipei 11217, Taiwan; 7 VGH Yang-Ming Genome Research Center, Taipei 11221, Taiwan

**Keywords:** circulating tumor cells, metastasis, CTC enrichment and enumeration

## Abstract

Circulating tumor cells (CTCs) can be detected in the blood of different types of early or advanced cancer using immunology-based assays or nucleic acid methods. The detection and quantification of CTCs has significant clinical utility in the prognosis of metastatic breast, prostate, and colorectal cancers. CTCs are a heterogeneous population of cells and often different from those of their respective primary tumor. Understanding the biology of CTCs may provide useful predictive information for the selection of the most appropriate treatment. Therefore, CTC detection and characterization could become a valuable tool to refine prognosis and serve as a “real-time biopsy” and has the potential to guide precision cancer therapies, monitor cancer treatment, and investigate the process of metastasis.

## 1. Introduction

Circulating tumor cells (CTCs) were first observed in the late 19th century [1]. Methods for the detection of CTCs in the peripheral blood of patients with epithelial tumors (including breast [2,3], colorectal [4,5], prostate [6] and lung [7]) have recently been developed. Since CTCs do not exist in patients without malignancy and have been detected in patients with almost all cancer types, CTCs are highly relevant for studying the biology of early metastatic spread and are used to diagnose patients with metastases [8]. Most studies demonstrate the enumeration of CTCs in patients with advanced stages of cancer, but an increasing number of publications report CTCs in patients at earlier disease stages [9,10]. The number of CTCs provides meaningful, real-time information on the clinical behavior of many tumors [2,5,6,11,12,13] and could additionally predict clinical outcome in patients with metastatic cancers [2,5,6].

CTCs most likely play a crucial role in mediating metastasis [14]. Metastasis is a multistep process caused by the dissociation of malignant cells from the primary tumor, their dissemination into circulation, and growth at distant sites. Tumor cells invade locally through the extracellular matrix and then intravasate into the lumina of blood vessels; as a result, CTCs travel through the bloodstream or the lymphatic system. The cells then arrest at distant organ sites and extravasate into distant tissues. Tumor cells survive in foreign microenvironments and then proliferate at metastatic sites, thereby generating macroscopic, clinically detectable neoplastic growths [15,16].

CTCs can be enriched and detected via various technologies that take advantage of their physical and biological properties. In recent years, major technological advances have been developed to identify CTCs, including cytometric methods [4,17,18], PCR-based assays [19,20,21], size-exclusion methods [22], and the CellSearch™ system (Veridex, Raritan, NJ, USA) [2,5,6]. The CTC number could provide information on the patient’s prognosis and therapeutic targets. It is unclear which method is optimal to quantify and characterize the biology and regulation of CTCs with respect to metastasis. This review focuses on the biology, enrichment/capture methods, and clinical potential of CTCs.

## 2. Biology of Circulating Tumor Cells (CTCs)

CTCs were previously believed to be rare among the population of primary tumor cells [23]. It has recently been reported that CTCs represent a heterogeneous pool of tumor cells [24,25,26]. Single-cell profiling of CTCs isolated from patients showed transcriptional heterogeneity [26]. Like the primary tumor cells, CTCs also express epithelial markers such as epithelial cell adhesion molecule (EpCAM) or certain cytokeratins (CKs). However, during the epithelial mesenchymal transition (EMT), some CTCs acquire altered phenotypes that may be related to tumor aggressiveness. Several groups have investigated this phenomenon in peripheral blood using various EMT-associated markers. Raimondi and colleagues [27] found that fibronectin and/or vimentin are expressed more frequently in CK^−^ than in CK^+^ blood samples. Armstrong and colleagues [28] found that the majority of CTCs in patients with metastatic prostate and metastatic breast cancer co-express epithelial proteins (EpCAM, CK, E-cadherin) and mesenchymal proteins, including vimentin and N-cadherin. Yu *et al.* [29] demonstrated that mesenchymal features are highly enriched in CTCs. These findings indicate that a pool of CTCs may have a partial mesenchymal phenotype. Chao *et al.* [30] demonstrated that tail vein injections of mesenchymal MDA-MB-231 cells into secondary organ microenvironments can induce the re-expression of E-cadherin and consequently MET. Bonnomet *et al.* [31] reported a heterogeneous expression pattern of vimentin in MDA-MB-468 xenografts and clinical human invasive breast tumor specimens. This suggests that vimentin-positive CTCs might have undergone MET to form vimentin-negative macrometastases. CTCs express a strong epithelial gene program and epithelial-type cells promote tumor colonization and proliferation [32].

EMT is associated with stemness properties. The induction of EMT results in expression of stem cell markers and gain of epithelial stem cell properties in cancer cells [33]. EMT also contributes to drug resistance [34]. Accordingly, subpopulations of CTCs may demonstrate a stem cell-like phenotype that might contribute to the metastatic spread of primary tumors and resistance to conventional chemotherapy. Recent experimental evidence in a variety of tumors strongly supports the ideal that cancer stem cell that drive tumorigenesis and metastasis [35,36]. Aldehyde dehydrogenase isoform 1 (ALDH1) is a functional marker of cancer stem cells [37,38]. Aktas *et al.* [39] observed that 69% of CTC-positive patients are also positive for *ALDH1* expression; this subset of ALDH1-positive patients more likely to respond to chemotherapy. CD44 has also been reported as a cell surface marker for breast, prostate and colon cancer stem cell [40,41,42]. Li *et al.* [43] demonstrated that patients with CD44-positive CTCs are more likely to develop metastasis and recurrence than patients with CD44-negative CTCs. CD44-positive CTC counts are higher in recurrent than non-recurrent patients [43]. Gradilone *et al.* [44] demonstrated that CTCs in 86% of metastatic breast cancer (MBC) patients expressed one or more multidrug resistance-related proteins (MRPs); patients with MRP-positive CTCs had shorter times to progression. These findings suggest that a pool of CTCs may exhibit some stem cell-like properties that contribute to metastatic spread and drug resistance.

Evidence suggests that CTCs are a highly heterogeneous pool of tumor cells [24]. CTCs may be viable or apoptotic; a portion of CTCs are cell-cycle-arrested and are chemo/radio-resistant. These non-cycling CTCs, referred to as dormant tumor cells, can be resistant to therapy and are often responsible for cancer relapse [45,46,47]. Because CTC count is an effective indicator of patient survival, their viability may be able to predict a patient’s response to therapies. Epithelial immunospot assays (EPISPOT) can directly detect proteins secreted from viable tumor cells [48]. Dying or dead CTCs that do not produce or secrete epithelial-associated proteins are not identified by this method. The EPISPOT assay has successfully identified metastatic breast cancer (mBC) cells cultured from blood and bone marrow using MUC1 and CK19 as marker proteins. The EPISPOT assay could be effective in evaluating more accurate CTC counts based on viability, but this test has not, to date, been evaluated in large-scale clinical trials or undergone formal commercial development.

## 3. CTC Detection and Quantification

CTCs circulate in peripheral blood at an extremely low frequency of approximately one CTC per 10^5^–10^7^ mononuclear cells in metastatic cancer patients; CTC numbers are even lower in non-metastatic cancer patients [49,50]. Since most cancers do not have specific markers, the enumeration of CTCs is limited. Current methods for detecting CTCs include enrichment and detection steps. The enrichment of CTCs is based on cell size, density, and positive immunoselection (e.g., epithelial cell adhesion molecule (EpCAM) [51] antibody-based enrichment of CTCs) or negative immunoselection (e.g., depletion of leukocytes by CD45 antibodies [52,53]). Methods of CTC detection are broadly divided into nucleic-acid based approaches (PCR targeting of various epithelial mRNAs, cytokeratins (CKs) [8] and EpCAM), immunology-based (immunocytochemistry with anti-CK or -EpCAM antibodies), and epithelial immunospot (EPISPOT) assays (detecting tumor-specific proteins).

### 3.1. Enrichment and Capture of CTCs

CTCs can be separated from blood cells according to their physical properties (Table 1). CTCs are ~20–30 μm in diameter, while blood cells measure ~8–12 μm [54]. Several devices have been developed based on cell filtration [55]. ISET^®^ (Rarecells Diagnostics, Paris, France) and ScreenCell^®^ (ScreenCell, Westford, MA, USA) utilize size to detect CTCs in the blood [54,56,57]. By exploiting the size difference, CTCs can also be isolated by centrifugal forces [58]. Because some epithelial markers are lost during EMT prior to metastasis, size-based enrichment has the advantages of higher capture efficiency and antigen-independent expression. However, the disadvantages of these systems include low CTC purity and potential leucocyte contamination. Additionally, smaller CTCs or fragments of CTCs could be lost. Ficoll-gradient enrichment is performed to separate mononuclear cells and CTCs (density < 1.077 g/mL) from other cells [59]. This method is based on cell density and can enrich most CTCs [60]. However, Ficoll enrichment of CTCs is largely due to loss of tumor cells that either migrate to the plasma layer or form aggregates at the bottom of the gradient.

**Table 1:** CTC enrichment approach.

Technology	Rationale	Platform	Product	Selected reference
Physical properties	Cell size Density	Physical filter Centrifugal force CTC-iChip Density gradient	ISET/RARECELL Early in development Early in development	[54,55,56,57] [58] [66] [59,60]
Antibody capture	Positive selection of Epithelial-specific antigen Negative depletion of leukocytes	CTC Chips Magnetic	Early in development MegSweeper (prototypes) CELLSEARCH CTC kit	[65] [64] [2,5,6]

Immuno-based separation is the most commonly used technique for CTC enrichment; this method relies on specific CTC markers that are detected by antibodies (Table 1). Epithelial markers are expressed on epithelial tumors but not on blood cells and have therefore been used to separate CTCs from blood cells. EpCAM and members of the family of CKs (CK8, CK18, and CK19) have been markers for positive selection in patients with carcinoma. Tumor-specific antigens are expressed at a higher level in cancer cells than normal ones. Her-2 [61] and epidermal growth factor receptor (EGFR) are tumor-associated markers for detecting CTCs. Tissue-specific antigens, including prostate-specific antigen (PSA) for prostate cancer, carcinoembryonic antigen (CEA) [62] for colon cancer and mammaglobin for breast cancer have a high specificity in cancer cells (Table 2). Negative selection for the antigens CD45 (expressed in leukocytes) and CD61 (expressed in megakaryocytes and platelets) [63] could avoid contamination by blood cells. MegSweeper can enrich CTCs and eliminate cells that are not bound to magnetic particles [64]. The major advantage of immune-based separation is that CTCs can be directly visualized and quantified without requiring cell lysis. Its limitations include cost and variability owing to the absence of standardized methods and reagents; furthermore, the loss of epithelial antigens by CTCs during EMT cannot be assessed by positive selection.

**Table 2:** Positive/negative markers for enrichment and detection of CTCs in different tumor types.

Tumor Types	Positive Markers	Negative Makers
Breast	EpCAM/CK Her-2 Mammaglobin MUC-1	CD45/CD61
Prostate PSA	EpCAM/CK	CD45
Colon	EpCAM/CK CEA	CD45
Lung	EpCAM/CK	CD45

A microfluidic platform (CTC chip) capable of separating CTCs from whole blood samples has also been developed. The CTC chip works by mediating the interaction of target CTCs with EpCAM-coated microspots, under precisely controlled laminar flow conditions [65]. Using this device, high numbers of CK-positive CTCs have been reported in nearly all tested patients with lung, prostate, pancreatic, breast, and colon cancers. Recently, tumor-antigen-independent microfluidic technology (CTC-iChip) was developed and uses deterministic lateral displacement, inertial focusing and magnetophoresis to sort CTCs with a marker-free approach [66]. CTC-iChip has the advantages of avoiding the loss of epithelial antigens during EMT.

### 3.2. Detection of CTCs

#### 3.2.1. Nucleic Acid-Based Methods

Specific DNA or mRNA markers are used to identify CTCs in nucleic acid-based methods (Table 3). These genes may encode tissue-, organ-, or tumor-specific proteins in cancer cells. Epithelial-specific genes such as CK and EpCAM, which are normally absent in circulation, are widely used as tissue-specific markers. Organ-specific markers, including the genes encoding CEA, PSA [67], mammaglobin [68], and MUC-1 [69], are employed to detect CTCs. Tumor-specific markers such as the EGFR and HER-2 genes have also been utilized to detect CTCs [70]. Indeed, the presence of tumor/organ-specific markers in the blood is correlated with higher levels of metastasis and worse prognosis.

Nucleic acid-based CTC detection is considered to be more sensitive than protein-based approaches (one CTC out of 10^6^–10^7^ of blood cells) [71]. Specificity, however, is low due to numerous potential false-positive results, including free DNA molecules released by necrotic or apoptotic CTCs, target genes expressed in other non-malignant cells during inflammation, invasive diagnostic biopsies, or tumor resection surgeries. Another important limitation of PCR-based methods is that CTCs cannot be isolated and have to be lysed before PCR, which prevents their direct observation, enumeration, and further analysis. Moreover, nucleic acid-based CTC detection does not provide a precise assessment of the number of tumor cells present in the sample making overall assessment for a multi-site study or comparisons of data across studies difficult.

**Table 3:** Advantages and disadvantages of CTC detection approach.

Platform	Rationale	Advantages	Disadvantages
Nucleic acid method	Epithelial/Tumor/Tissue-specific antigen	High sensitivity Small blood volume required Rapid	Cells need to be lysed Low specificity CTC without Epithelial marker could not be detected
Flow cytometery	Positive selection of Epithelial-specific antigen Negative depletion of leukocytes	Quantitative and multiparameter High specificity Potential to sort CTCs	Limited sensitivity Requirement for large sample volume CTC without epithelial marker could not be detected
CellSearch (Veridex)	Positive selection of EpCAM/CK Negative depletion of CD45	High sensitivity and specificity Highly reproducible Commercially available Only assay with FDA approval	CTC without epithelial marker could not be detected
CTC Chip	Positive selection of EpCAM/CK	High sensitivity and specificity Potential to recover CTCs for additional characterization	Technology is not commercially available

#### 3.2.2. Immunological-Based Assay

Immunology-based techniques utilize labeled antibodies directed against epithelial or tumor-associated antigens along with automated digital microscopy or flow cytometry to identify and quantify CTCs. Immunological methods are the most common of the approaches and are effective for both detection and isolation of CTCs. Many antigens have been used for this approach, including EpCAM and different subtypes of CKs. However, not all CTCs express these markers, possibly a consequence of the EMT process and likely lead to false-negative results. Rao *et al.* [72] demonstrated that EpCAM expression was approximately ten-fold lower in CTCs than in primary and metastatic tissues. Several organ- or tumor-specific markers, including CEA, EGFR, PSA, HER-2, and MUC-1, have also been applied in antibody-based detection and isolation of CTCs [73,74,75].

CTC detection by immunocytochemistry followed by fluorescence microscopy enables direct visualization of antibody-labeled target cells. The CD45 marker is employed to rule out white blood cells and increase detection specificity. A CTC is often defined as a CK+/CD45-/DAPI+ intact cell [14]. The nuclear dye DAPI is used to exclude cell fragments and debris false-positive may occur using CK as a marker. Circulating epithelial cells may be recognized by CK-positive selection while trauma or inflammation within the body. Immunocytochemistry is considered the most reliable and specific method for CTC detection; however, the number of cells detected is low and it is not a practically viable method.

Flow cytometry, including fluorescence-activated cell sorting, is often employed for the detection, enumeration, and separation of immunofluorescently labeled CTCs [76]. Flow-based assays have high specificity because the capabilities for analysis of multiple parameters by cell-by-cell and easy approach for CTC isolation and analysis. The sensitivity is around 10*^−^*^4^ to 10*^−^*^5^ in circulating mono-nucleated cells while CTCs detection in peripheral blood. Blood volume needs to be increased to accurately and reproducibly detect and enumerate very small numbers of CTCs in flow cytometry-based CTC assays. It is estimated that, if the frequency of CTCs is 10^−6^, at least 1.5 × 10^7^ nucleated cell events must be acquired by a flow cytometer [77]. About 12 mL of whole blood would have to be assessed if the cell event was elevated for lower frequency at one CTC in 10^7^ leukocytes [78].

Racilla and colleagues developed a highly sensitive assay combining immunomagnetic enrichment with flow cytometry and immunocytochemical analysis to detect, enumerate, and characterize carcinoma cells in the blood [79]. Based on this approach, CellSearch^®^ system (Veridex, Raritan, NJ, USA) is developed. CellSearch^®^ system (Veridex, Raritan, NJ, USA) is an automated immunomagnetic enrichment and staining system for CTCs which is the only clinically approved assay for CTC detection. This system uses negative immunomagnetic selection against CD45, and positive immunomagnetic selection against EpCAM followed by CK staining of CTCs in 7.5 mL blood samples. The system has a sensitivity of detection of approximately 2 CTC per 7.5 mL of whole blood and appears to provide clinically useful information on the prognosis of patients with metastatic breast, colon and prostate cancer [2,5,6]. The advantages of this system include high sensitivity specificity, reproducibility, as well as automation [80]. CellSearch provides a precise assessment of the acute number of tumor cells present in the 7.5 mL sample. Limitation is that multiple enrichments may result in loss of CTCs; furthermore, the loss of epithelial antigens by CTCs during EMT cannot be assessed by positive selection.

## 4. Clinical Manifestation of CTCs

**Figure 1:**
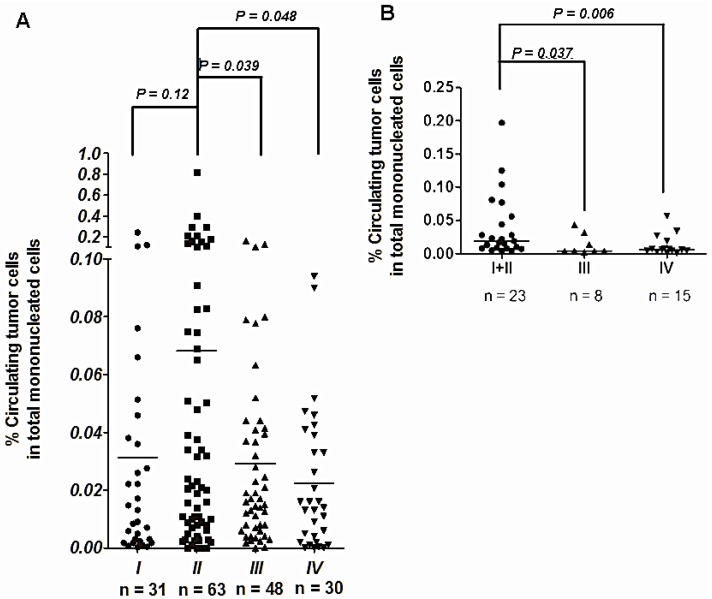
The number of circulating tumor cells in CRC and lung cancer patients (**A**) Cells isolated from fresh blood drawn from the mesenteric vein of CRC patients were subjected to surface marker staining and FACS analysis. The percentage of CTCs was quantified by counting CD45^dim^ ESA^+^ cells among all mononucleated cells. The average number of CTCs is higher in patients with stage II CRC, than in patients with stage I, III, and IV; (**B**) Fresh blood samples from the forearm vein of lung cancer patients were subjected to surface marker staining and FACS analysis. CTCs are calculated in the lung cancer patients of different clinical stages. The number of CTCs detected from forearm vein of clinical stage I/II lung cancer patients are higher than those of stages III and IV.

Since CTCs play crucial roles in metastasis and drug resistance, it is important to understand their clinical manifestation in the early stages of diseases. In breast cancer, Franken *et al.* [81] detected ≥1 CTC in 30 mL blood in 16% of patients in stage I, 18% in stage II, and 31% in stage III. The CTC number positively relates with disease stage. Among patients with at least one CTC, 16 (21.1%) developed a recurrence, whereas 11.6% of patients with no CTCs developed a recurrence. Presence of CTC in breast cancer patients before undergoing surgery is associated with disease-free survival (DFS). [82]. In GI cancers, the liver may act as a physical blood filter for CTCs released from the primary tumor. CTCs are found at a higher rate (*p* = 0.01) and a higher count (*p* = 0.006) in the mesenteric venous blood (MVBC) than in central venous blood (internal jugular or subclavian vein) [83]. Tseng *et al.* reported that the number of mesenteric CTCs was 1–2 orders of magnitude higher than the number of forearm CTCs obtained from the same colorectal cancer (CRC) patient. The percentage of mesenteric CTCs in the total mononucleated cell population ranged from 0 to 0.815% (Figure 1). The number of mesenteric CTCs obtained from early-stage patients was higher than that obtained from patients in stages III and IV of CRC [84]. In lung cancer patients, forearm CTCs are also highest in stage I and II patients, similar to mesenteric CTCs in CRC cases and the percentage of forearm CTCs in the total mononucleated cell population ranged from 0 to 0.19% (Figure 1). The higher number of CTCs in stage I and II lung cancer patients may be due to primary lung cancer cells drain directly to the systemic (forearm vein) circulation without passing through the portal (liver) or pulmonary circulation. Taken together, these results strongly suggest that only CTC measurements made from the direct venous drain of the primary tumor reflect the level of intravasation of primary cancer cells and therefore the risk of metastasis.

## 5. Clinical Application of CTCs

There is a great interest in developing methods to extract and analyze CTCs for the diagnosis, prognosis, and clinical management of cancer patients. Utilizing multiple blood tests, the number of CTCs could provide information on patient prognosis, the likelihood of cancer relapse, and predictions regarding drug resistance.

### 5.1. CTC Enumeration in Patients with Early-Stage Disease

New prognosis tools in the setting of early-stage cancer have the potential to improve patient quality of life and enhance clinical decision making. In its current state, CTC detection may not yet be a well-established indicator for cancer diagnosis. A few studies suggest that CTC number may have a potential application in prognosis in early-stage disease. CTCs are found in ~20%–40% of patients with early-stage breast cancer according to PCR-based assays and ~10% of early-stage patients according to the CellSearch system [85]. CTC frequency is negatively correlated with outcome with respect to both progression-free (PFS) and overall survival (OS), independent of nodal status or adjuvant therapy [86,87]. In early-stage CRC, a few studies have observed CTCs in ~10%–20% of patients based on a PCR-based assay for CEA/CK20 and the CellSearch system. The median number of detected CTCs was one (range, zero to four) in 7.5 mL of peripheral blood [88]. Due to the different site used for CTC detection, CTC levels did not correlate with DFS or OS. Furthermore, there were no significant correlations between CTC levels and grade of differentiation, CEA levels, and lactate dehydrogenase levels. In contrast, CTC enumeration in a tumor’s draining vein is associated with lymph node metastasis and hepatic metastasis. These early reports demonstrate the feasibility and potential prognostic value of CTCs for CRC.

### 5.2. CTCs’ Enumeration in Patients with Metastatic Disease

In recent years, CTC enumeration has been established in many prospective studies as a prognostic marker for metastatic colorectal cancer (mCRC), breast cancer (mBC), prostate cancer (mPC), and lung cancer. Early studies reported that ~60%–70% of mBC patients have at least 2 CTCs, whereas CTCs are very rarely observed in normal control subjects using the CellSearch system [2,89]. These studies show significant correlations between CTC count at the time of diagnosis and the patient’s prognosis. Statistically, patients with ≥5CTCs at baseline had poorer PFS and OS than patients with ≤5 CTCs in a 7.5 mL blood sample [2]. In patients with mCRC, Cohen *et al.* [5] reported that median PFS and OS rates were approximately twice as high for patients with few CTCs (<3 CTCs/7.5 mL blood) relative to patients with elevated CTCs (≥3 CTCs/7.5 mL blood) as estimated at baseline. Evidence suggests similar results for mPC with a threshold of 5 CTCs [54]. After chemotherapy and/or radiotherapy, the CTC level was inversely correlated with PFS and OS in mBC, mPC and mCRC. In some cases, CTC analysis was better for predicting treatment response than commonly employed methods such as radiological assessment (for breast cancer) and measurement of PSA (for prostate cancer). Taken together, CTC enumeration can be a useful prognostic biomarker for PFS or OS in metastatic cancers.

### 5.3. Circulating Tumor Cell Enumeration as an Indicator of Drug Response and Therapeutically Targets

Evidence suggests a strong correlation between CTC level and PFS and OS, indicating that CTCs are a good therapeutic indicator for mBC, mPC and mCRC. Numerous studies suggest that CTCs can be used for risk stratification and monitoring therapeutic efficacy. Many interventional trials have been specifically designed to demonstrate that CTC enumeration and characterization may improve the management of breast cancer patients. In mBC, patients with ≥5 CTCs/7.5 mL at 3–5 weeks following chemo- and hormone-therapies have a shorter PFS than patients with <5 CTCs/7.5 mL [90]; however, there is a strong correlation between CTC counts and radiographic disease progression in patients treated with chemo- and hormone-therapies [90]. Therefore, CTC enumeration could be used as an adjunct to standard methods for monitoring disease status in mBC.

CTC analyses have been incorporated into phase I and II clinical trials for the development of novel targeted therapies. In the development of Pertuzumab and Erlotinib for advanced non-small cell lung cancer [91], patients with ≥5 CTCs/7.5 mL at baseline were found to be significantly more likely to respond than patients with <5 CTCs/7.5 mL at baseline. Furthermore, CTC counts were associated with positive treatment response; decreased CTC counts upon treatment were associated with FDG-PET treatment and longer PFS. For castration-resistant prostate cancer, CTC counts are prognostic pre-therapy. Changes in the CTC number post-therapy are predictive of survival. In a phase II study of abiraterone for castration-resistant prostate cancer, CTC counts of 5 cells/7.5 mL were observed in 70% to 79% of subjects. Of the patients treated with abiraterone, 34% to 41% shifted to the more favorable subgroup with <5 cells/7.5 mL, providing evidence for drug activity in the patients [92,93]. In a phase III study, abiraterone was shown to improve OS and ~50% of patients were converted from having >5 cells/7.5 mL to <5 cells/7.5 mL [94]. In contrast, the phase III Southwest Oncology Group (SWOG) S0500 clinical trial showed that switching chemotherapy after one cycle based on elevated circulating tumor cell levels did not improve PFS or OS in mBC. This study confirmed that patients who have low numbers of CTCs before starting chemotherapy have a much better survival. Patients in whom CTCs remained elevated after one cycle of chemotherapy had substantially worse survival [95]. Thus, CTC quantification and characterization may be a potential biomarker for anti-cancer therapies and have a role in the clinical development of novel therapeutics.

## 6. Perspectives

CTC identification and characterization is meaningful for the interpretation of metastatic cancers (breast, prostate, colon, and lung cancer). The promise of CTC research in the early stages of cancer is largely unmet, requiring more sensitive and reproducible technologies. The “real-time biopsy” potential of CTCs is a key area for further intensive research and appropriate animal models provide the foundation for studies of the molecular regulation of CTCs in cancer and CTCs as biomarkers for therapeutic efficacy. The envisioned future is one in which a simple blood test will permit molecular tumor characterization, identification of treatment targets, and aid in the selection of the most appropriate targeted therapy from an armamentarium of agents.

## 7. Conclusions

The number of CTCs has a potential be an effective prognostic and predictive biomarker, which could assess therapeutic responses of metastatic disease in several cancers. The detection of CTCs in early stage cancer needs the further improvement of CTC assays. Since the heterogeneity of CTCs, the assays used to detect CTCs need tumor-specific rather than one technology for all cancer types. In conclusions, the better understand of the biology and clinical meaning of CTCs will help to improve CTC assays and further to apply in clinical utility.

## Abbreviations

CTC: Circulating tumor cell
CK: cytokeratin
EpCAM: Epithelial cell adhesion molecule
EMT: epithelial mesenchymal transition
PFS: Progression-free survival
OS: Overall survival
